# Correction: Multi-scale Inference of Interaction Rules in Animal Groups Using Bayesian Model Selection

**DOI:** 10.1371/annotation/f490031b-2e94-42c8-8c10-4e316a7435be

**Published:** 2012-03-06

**Authors:** Richard P. Mann, Andrea Perna, Daniel Strömbom, Roman Garnett, James E. Herbert-Read, David J. T. Sumpter, Ashley J. W. Ward

A coding flaw has been found in the software described in this article. The authors are determining if the flaw influences the findings of the paper. Until that determination is complete we caution readers against using the analysis program. A further update will be provided once the extent of this error is known. 

